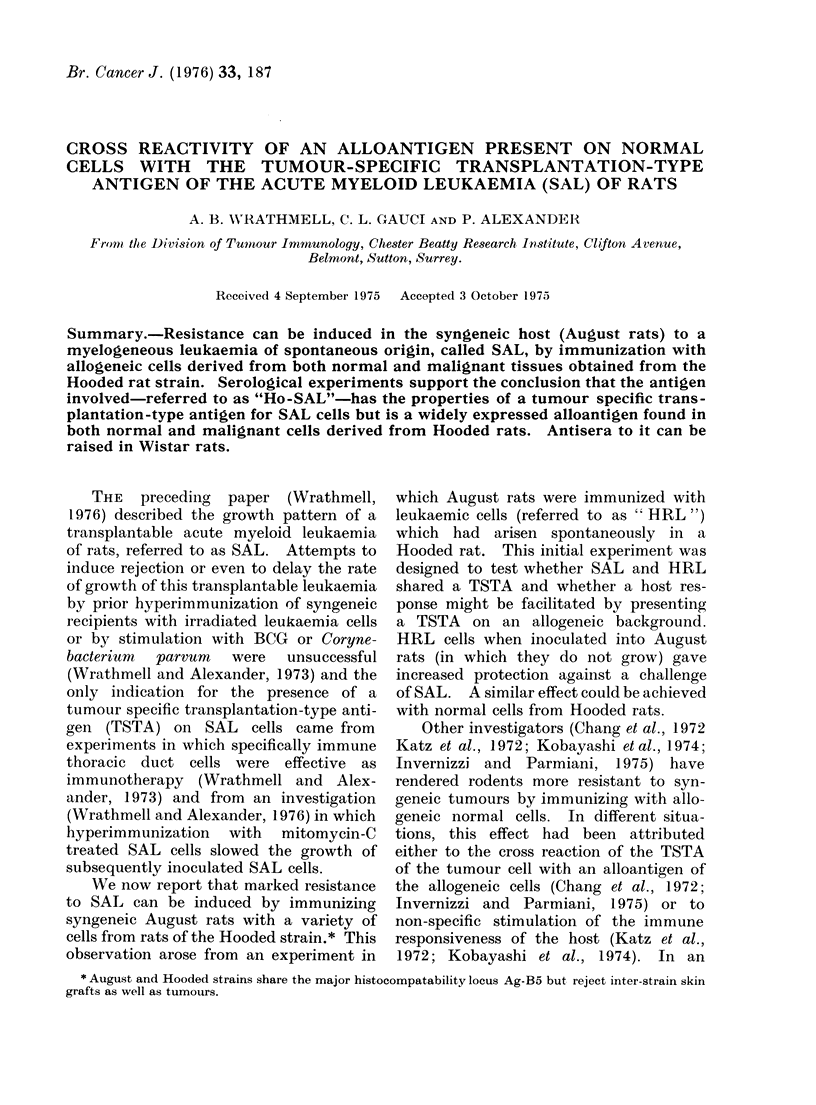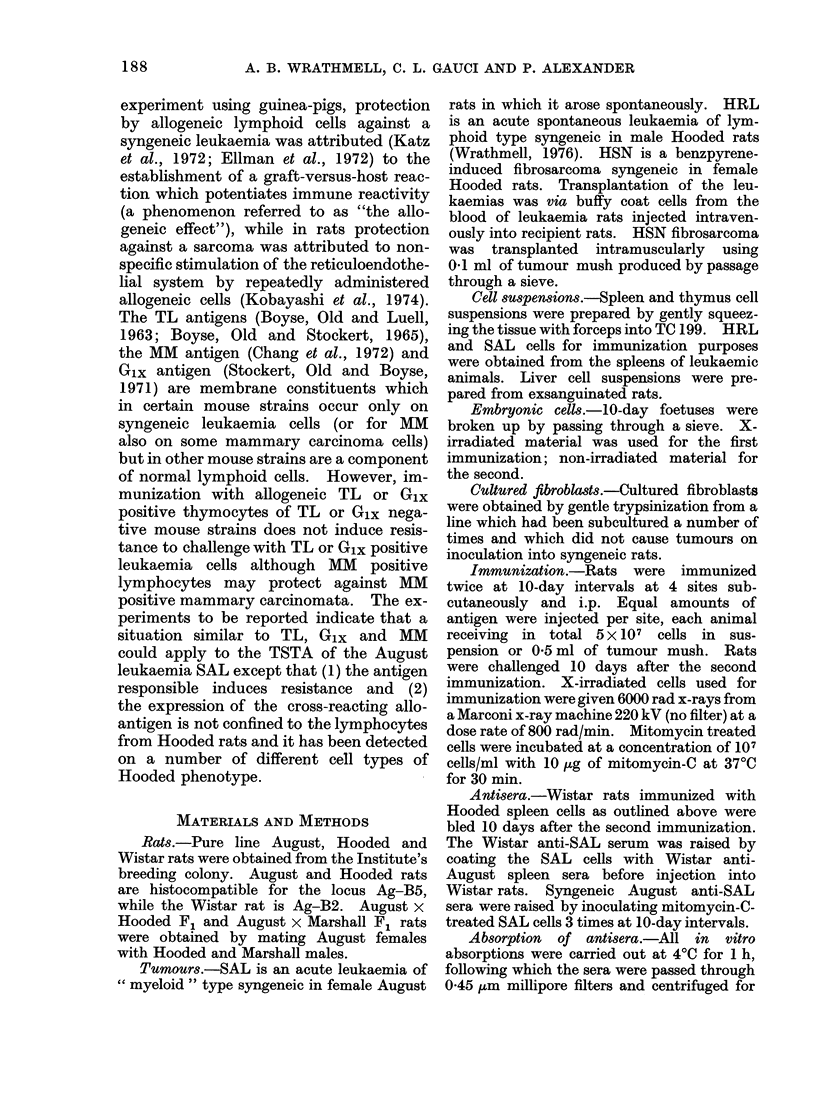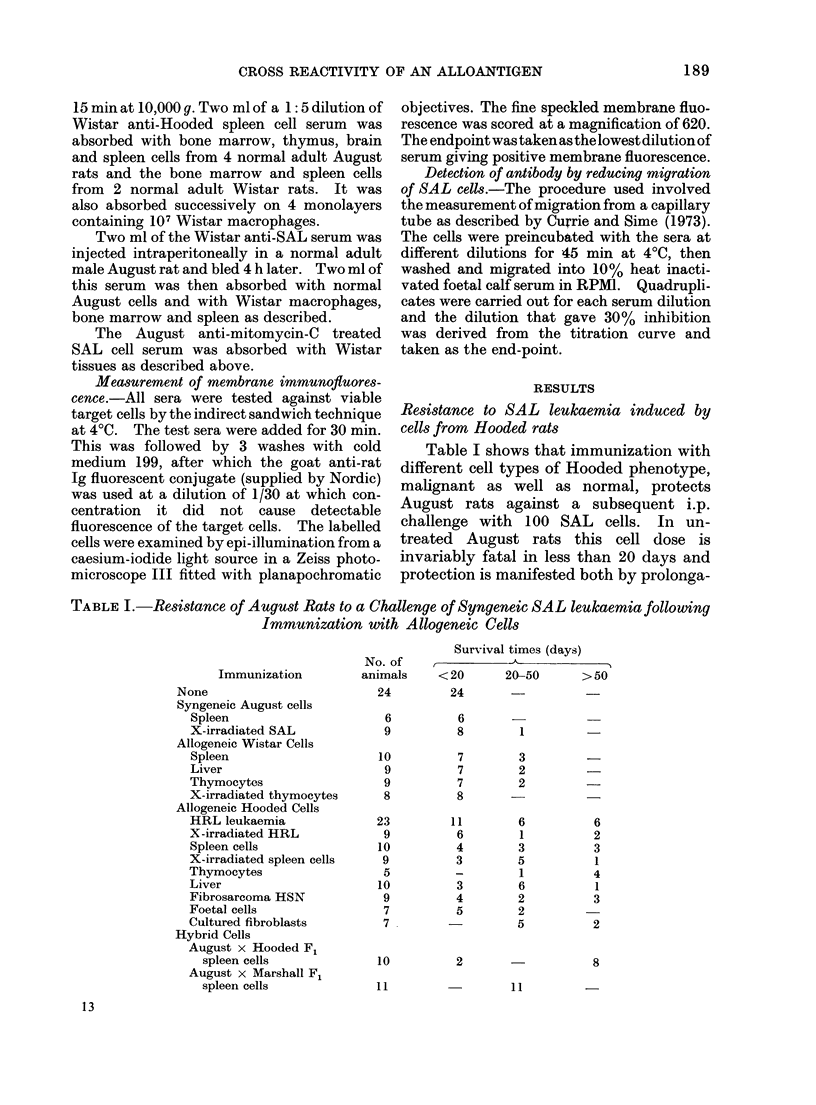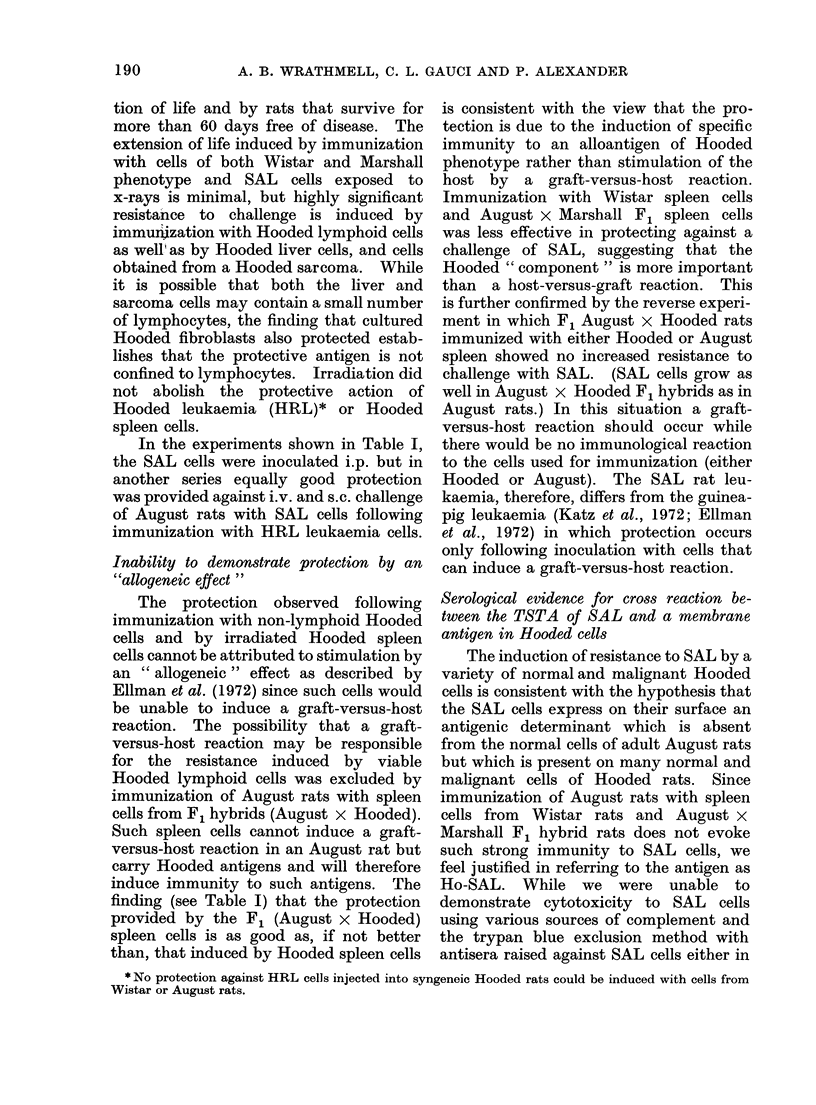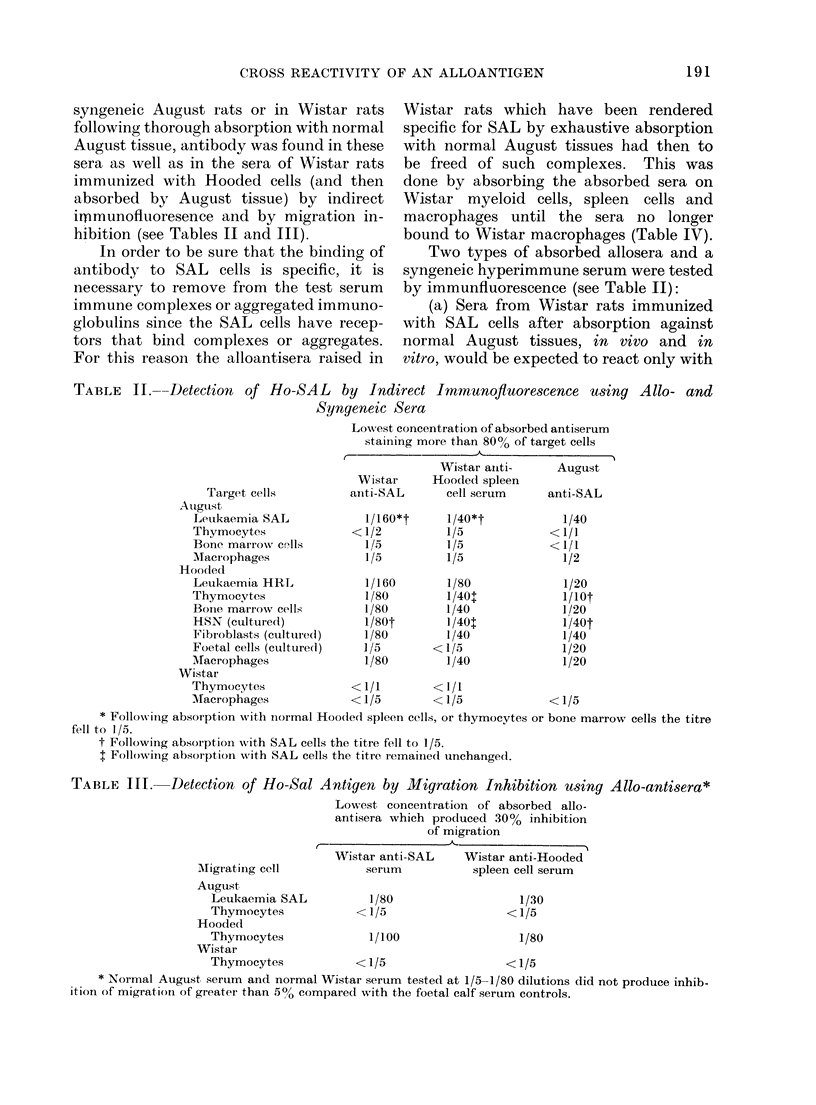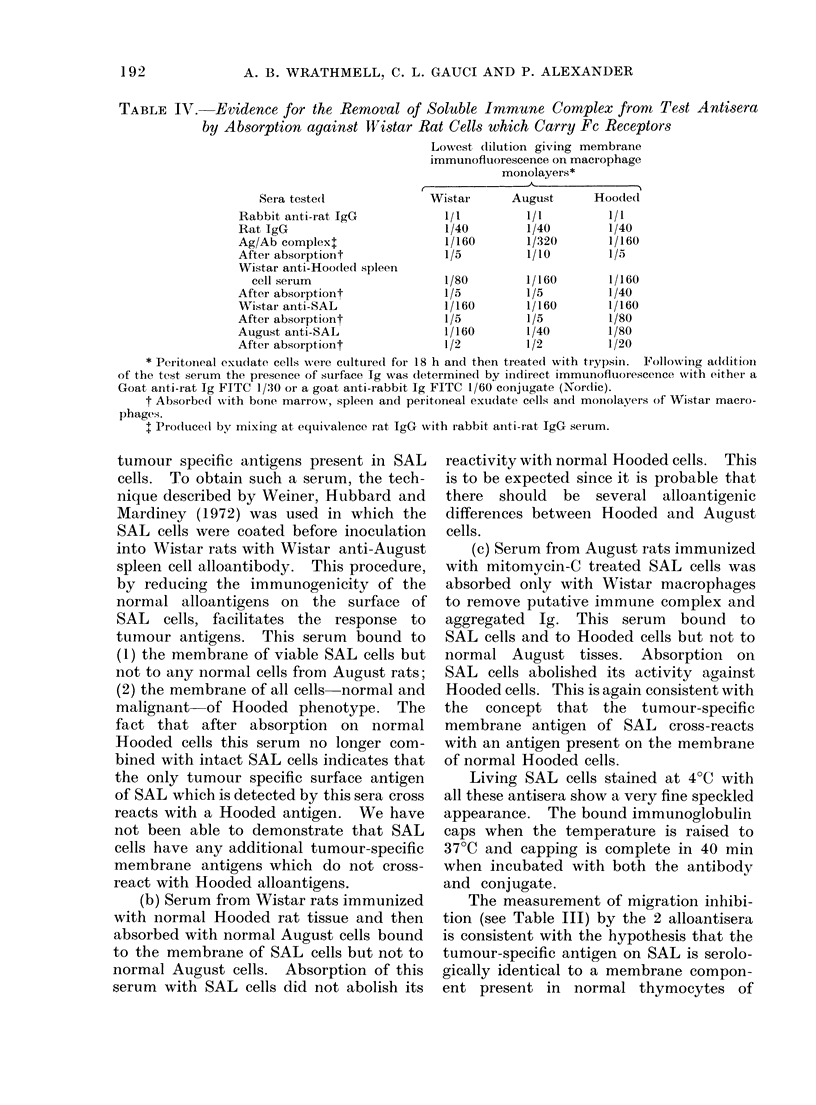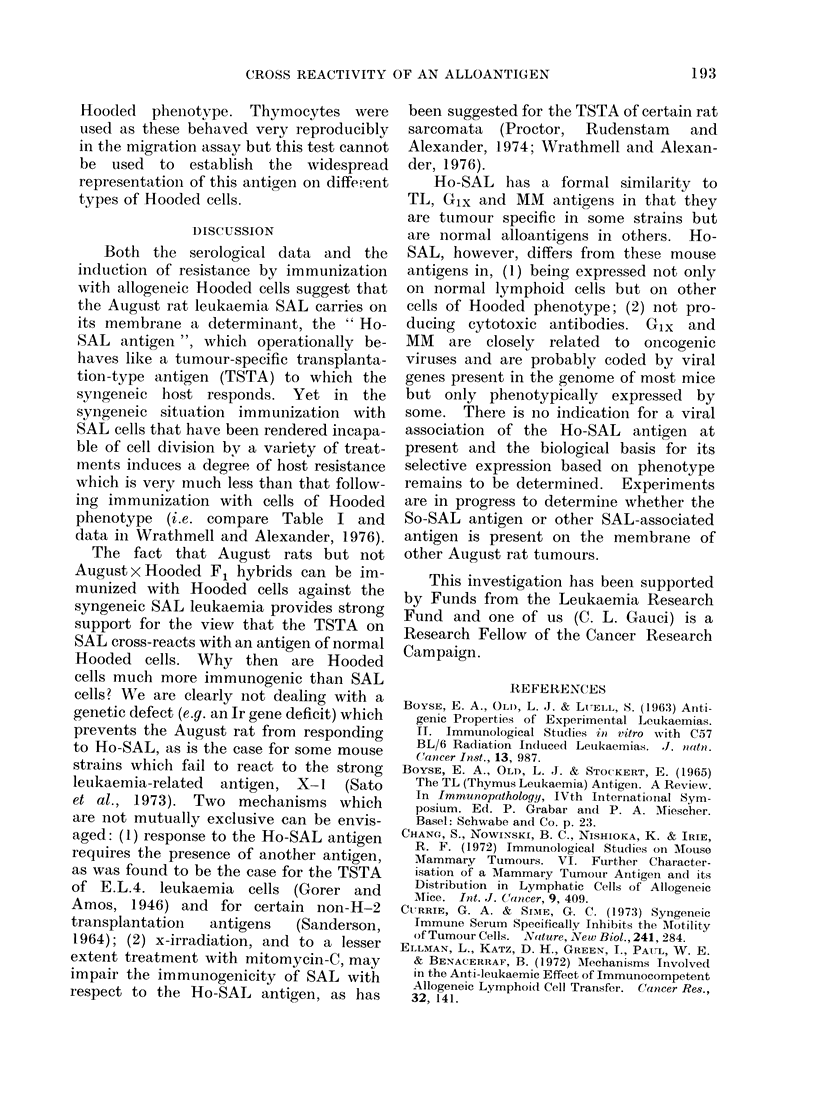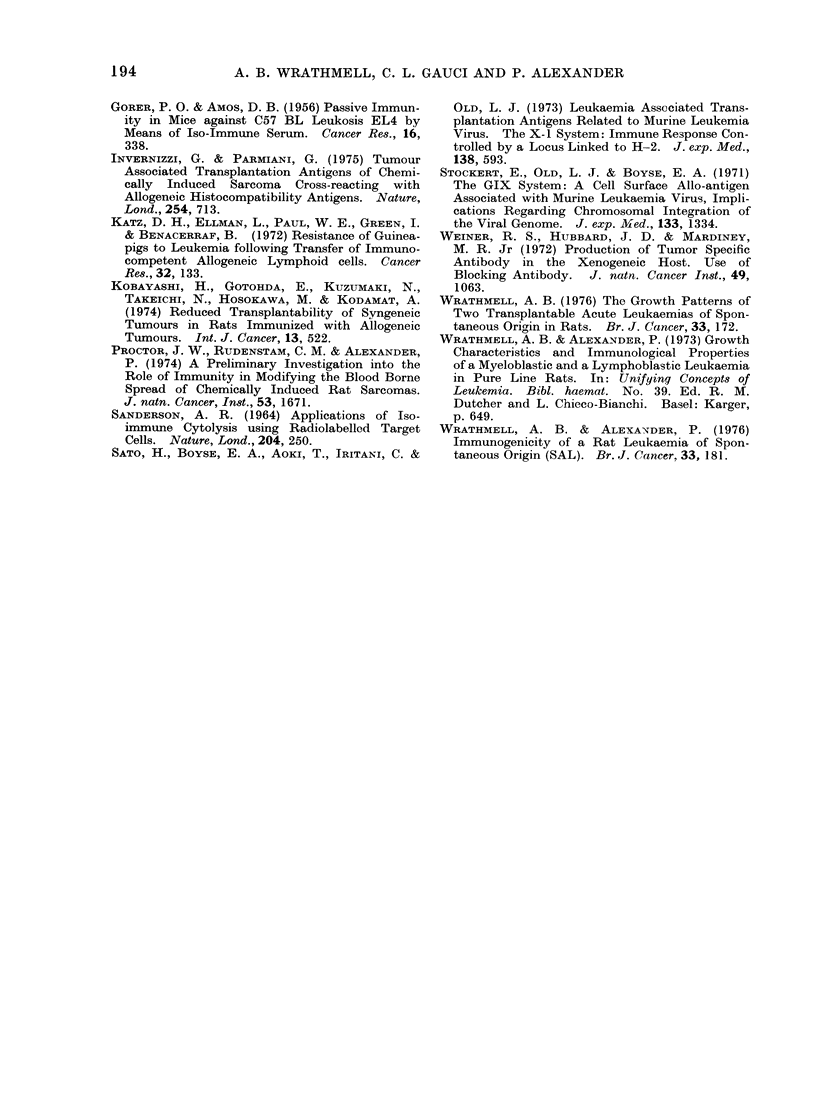# Cross reactivity of an alloantigen present on normal cells with the tumour-specific transplantation-type antigen of the acute myeloid leukaemia (SAL) of rats.

**DOI:** 10.1038/bjc.1976.24

**Published:** 1976-02

**Authors:** A. B. Wrathmell, C. L. Gauci, P. Alexander

## Abstract

Resistance can be induced in the syngeneic host (August rats) to a myelogeneous leukaemia of spontaneous origin, called SAL, by immunization with allogeneic cells derived form both normal and malignant tissues obtained from the Hooded rat strain. Serological experiments support the conclusion that the antigen involved-referred to as "Ho-SAL"-has the properties of a tumour specific transplantation-type antigen for SAL cells but is a widely expressed alloantigen found in both normal and malignant cells derived from Hooded rats. Antisera to it can be raised in Wistar rats.


Br. Cancer J. (1976) 33, 187

### CROSS REACTIVITY OF AN ALLOANTIGEN PRESENT ON NORMAL CELLS WITH THE TUMOUR-SPECIFIC TRANSPLANTATION-TYPE ANTIGEN OF THE ACUTE MYELOID LEUKAEMIA (SAL) OF RATS

A. B. WRATHMELL, C. L. GAUCI AND P. ALEXANDER

*From the Division of Tumour Immunology, Chester Beatty Research Institute, Clifton Avenue, Belmont, Sutton, Surrey.*

Received 4 September 1975 Accepted 3 October 1975

**Summary.—Resistance can be induced in the syngeneic host (August rats) to a myelogeneous leukaemia of spontaneous origin, called SAL, by immunization with allogeneic cells derived from both normal and malignant tissues obtained from the Hooded rat strain. Serological experiments support the conclusion that the antigen involved—referred to as "Ho-SAL"—has the properties of a tumour specific transplantation-type antigen for SAL cells but is a widely expressed alloantigen found in both normal and malignant cells derived from Hooded rats. Antisera to it can be raised in Wistar rats.**

THE preceding paper (Wrathmell, 1976) described the growth pattern of a transplantable acute myeloid leukaemia of rats, referred to as SAL. Attempts to induce rejection or even to delay the rate of growth of this transplantable leukaemia by prior hyperimmunization of syngeneic recipients with irradiated leukaemia cells or by stimulation with BCG or *Corynebacterium parvum* were unsuccessful (Wrathmell and Alexander, 1973) and the only indication for the presence of a tumour specific transplantation-type antigen (TSTA) on SAL cells came from experiments in which specifically immune thoracic duct cells were effective as immunotherapy (Wrathmell and Alexander, 1973) and from an investigation (Wrathmell and Alexander, 1976) in which hyperimmunization with mitomycin-C treated SAL cells slowed the growth of subsequently inoculated SAL cells.

We now report that marked resistance to SAL can be induced by immunizing syngeneic August rats with a variety of cells from rats of the Hooded strain.* This observation arose from an experiment in which August rats were immunized with leukaemic cells (referred to as "HRL") which had arisen spontaneously in a Hooded rat. This initial experiment was designed to test whether SAL and HRL shared a TSTA and whether a host response might be facilitated by presenting a TSTA on an allogeneic background. HRL cells when inoculated into August rats (in which they do not grow) gave increased protection against a challenge of SAL. A similar effect could be achieved with normal cells from Hooded rats.

Other investigators (Chang *et al.*, 1972 Katz *et al.*, 1972; Kobayashi *et al.*, 1974; Invernizzi and Parmiani, 1975) have rendered rodents more resistant to syngeneic tumours by immunizing with allogeneic normal cells. In different situations, this effect had been attributed either to the cross reaction of the TSTA of the tumour cell with an alloantigen of the allogeneic cells (Chang *et al.*, 1972; Invernizzi and Parmiani, 1975) or to non-specific stimulation of the immune responsiveness of the host (Katz *et al.*, 1972; Kobayashi *et al.*, 1974). In an

* August and Hooded strains share the major histocompatability locus Ag-B5 but reject inter-strain skin grafts as well as tumours.

experiment using guinea-pigs, protection by allogeneic lymphoid cells against a syngeneic leukaemia was attributed (Katz *et al.*, 1972; Ellman *et al.*, 1972) to the establishment of a graft-versus-host reaction which potentiates immune reactivity (a phenomenon referred to as "the allogeneic effect"), while in rats protection against a sarcoma was attributed to non-specific stimulation of the reticuloendothelial system by repeatedly administered allogeneic cells (Kobayashi *et al.*, 1974). The TL antigens (Boyse, Old and Luell, 1963; Boyse, Old and Stockert, 1965), the MM antigen (Chang *et al.*, 1972) and $G_{IX}$ antigen (Stockert, Old and Boyse, 1971) are membrane constituents which in certain mouse strains occur only on syngeneic leukaemia cells (or for MM also on some mammary carcinoma cells) but in other mouse strains are a component of normal lymphoid cells. However, immunization with allogeneic TL or $G_{IX}$ positive thymocytes of TL or $G_{IX}$ negative mouse strains does not induce resistance to challenge with TL or $G_{IX}$ positive leukaemia cells although MM positive lymphocytes may protect against MM positive mammary carcinomata. The experiments to be reported indicate that a situation similar to TL, $G_{IX}$ and MM could apply to the TSTA of the August leukaemia SAL except that (1) the antigen responsible induces resistance and (2) the expression of the cross-reacting alloantigen is not confined to the lymphocytes from Hooded rats and it has been detected on a number of different cell types of Hooded phenotype.

### Materials and Methods

*Rats.*—Pure line August, Hooded and Wistar rats were obtained from the Institute's breeding colony. August and Hooded rats are histocompatible for the locus Ag–B5, while the Wistar rat is Ag–B2. August × Hooded $F_1$ and August × Marshall $F_1$ rats were obtained by mating August females with Hooded and Marshall males.

*Tumours.*—SAL is an acute leukaemia of " myeloid " type syngeneic in female August rats in which it arose spontaneously. HRL is an acute spontaneous leukaemia of lymphoid type syngeneic in male Hooded rats (Wrathmell, 1976). HSN is a benzpyrene-induced fibrosarcoma syngeneic in female Hooded rats. Transplantation of the leukaemias was *via* buffy coat cells from the blood of leukaemia rats injected intravenously into recipient rats. HSN fibrosarcoma was transplanted intramuscularly using 0·1 ml of tumour mush produced by passage through a sieve.

*Cell suspensions.*—Spleen and thymus cell suspensions were prepared by gently squeezing the tissue with forceps into TC 199. HRL and SAL cells for immunization purposes were obtained from the spleens of leukaemic animals. Liver cell suspensions were prepared from exsanguinated rats.

*Embryonic cells.*—10-day foetuses were broken up by passing through a sieve. X-irradiated material was used for the first immunization; non-irradiated material for the second.

*Cultured fibroblasts.*—Cultured fibroblasts were obtained by gentle trypsinization from a line which had been subcultured a number of times and which did not cause tumours on inoculation into syngeneic rats.

*Immunization.*—Rats were immunized twice at 10-day intervals at 4 sites subcutaneously and i.p. Equal amounts of antigen were injected per site, each animal receiving in total $5 \times 10^7$ cells in suspension or 0·5 ml of tumour mush. Rats were challenged 10 days after the second immunization. X-irradiated cells used for immunization were given 6000 rad x-rays from a Marconi x-ray machine 220 kV (no filter) at a dose rate of 800 rad/min. Mitomycin treated cells were incubated at a concentration of $10^7$ cells/ml with 10 $\mu$g of mitomycin-C at 37°C for 30 min.

*Antisera.*—Wistar rats immunized with Hooded spleen cells as outlined above were bled 10 days after the second immunization. The Wistar anti-SAL serum was raised by coating the SAL cells with Wistar anti-August spleen sera before injection into Wistar rats. Syngeneic August anti-SAL sera were raised by inoculating mitomycin-C-treated SAL cells 3 times at 10-day intervals.

*Absorption of antisera.*—All *in vitro* absorptions were carried out at 4°C for 1 h, following which the sera were passed through 0·45 $\mu$m millipore filters and centrifuged for

15 min at 10,000 $g$. Two ml of a 1 : 5 dilution of Wistar anti-Hooded spleen cell serum was absorbed with bone marrow, thymus, brain and spleen cells from 4 normal adult August rats and the bone marrow and spleen cells from 2 normal adult Wistar rats. It was also absorbed successively on 4 monolayers containing $10^7$ Wistar macrophages.

Two ml of the Wistar anti-SAL serum was injected intraperitoneally in a normal adult male August rat and bled 4 h later. Two ml of this serum was then absorbed with normal August cells and with Wistar macrophages, bone marrow and spleen as described.

The August anti-mitomycin-C treated SAL cell serum was absorbed with Wistar tissues as described above.

*Measurement of membrane immunofluorescence.*—All sera were tested against viable target cells by the indirect sandwich technique at 4°C. The test sera were added for 30 min. This was followed by 3 washes with cold medium 199, after which the goat anti-rat Ig fluorescent conjugate (supplied by Nordic) was used at a dilution of 1/30 at which concentration it did not cause detectable fluorescence of the target cells. The labelled cells were examined by epi-illumination from a caesium-iodide light source in a Zeiss photomicroscope III fitted with planapochromatic objectives. The fine speckled membrane fluorescence was scored at a magnification of 620. The endpoint was taken as the lowest dilution of serum giving positive membrane fluorescence.

*Detection of antibody by reducing migration of SAL cells.*—The procedure used involved the measurement of migration from a capillary tube as described by Currie and Sime (1973). The cells were preincubated with the sera at different dilutions for 45 min at 4°C, then washed and migrated into 10% heat inactivated foetal calf serum in RPMI. Quadruplicates were carried out for each serum dilution and the dilution that gave 30% inhibition was derived from the titration curve and taken as the end-point.

### RESULTS

### *Resistance to SAL leukaemia induced by cells from Hooded rats*

Table I shows that immunization with different cell types of Hooded phenotype, malignant as well as normal, protects August rats against a subsequent i.p. challenge with 100 SAL cells. In untreated August rats this cell dose is invariably fatal in less than 20 days and protection is manifested both by prolonga-

TABLE I.—*Resistance of August Rats to a Challenge of Syngeneic SAL leukaemia following Immunization with Allogeneic Cells*

| Immunization | No. of animals | Survival times (days) <20 | 20–50 | >50 |
|---|---|---|---|---|
| None | 24 | 24 | — | — |
| Syngeneic August cells | | | | |
| Spleen | 6 | 6 | — | — |
| X-irradiated SAL | 9 | 8 | 1 | — |
| Allogeneic Wistar Cells | | | | |
| Spleen | 10 | 7 | 3 | — |
| Liver | 9 | 7 | 2 | — |
| Thymocytes | 9 | 7 | 2 | — |
| X-irradiated thymocytes | 8 | 8 | — | — |
| Allogeneic Hooded Cells | | | | |
| HRL leukaemia | 23 | 11 | 6 | 6 |
| X-irradiated HRL | 9 | 6 | 1 | 2 |
| Spleen cells | 10 | 4 | 3 | 3 |
| X-irradiated spleen cells | 9 | 3 | 5 | 1 |
| Thymocytes | 5 | – | 1 | 4 |
| Liver | 10 | 3 | 6 | 1 |
| Fibrosarcoma HSN | 9 | 4 | 2 | 3 |
| Foetal cells | 7 | 5 | 2 | — |
| Cultured fibroblasts | 7 | — | 5 | 2 |
| Hybrid Cells | | | | |
| August × Hooded $F_1$ spleen cells | 10 | 2 | — | 8 |
| August × Marshall $F_1$ spleen cells | 11 | — | 11 | — |

tion of life and by rats that survive for more than 60 days free of disease. The extension of life induced by immunization with cells of both Wistar and Marshall phenotype and SAL cells exposed to x-rays is minimal, but highly significant resistance to challenge is induced by immunization with Hooded lymphoid cells as well as by Hooded liver cells, and cells obtained from a Hooded sarcoma. While it is possible that both the liver and sarcoma cells may contain a small number of lymphocytes, the finding that cultured Hooded fibroblasts also protected establishes that the protective antigen is not confined to lymphocytes. Irradiation did not abolish the protective action of Hooded leukaemia (HRL)* or Hooded spleen cells.

In the experiments shown in Table I, the SAL cells were inoculated i.p. but in another series equally good protection was provided against i.v. and s.c. challenge of August rats with SAL cells following immunization with HRL leukaemia cells.

*Inability to demonstrate protection by an "allogeneic effect"*

The protection observed following immunization with non-lymphoid Hooded cells and by irradiated Hooded spleen cells cannot be attributed to stimulation by an "allogeneic" effect as described by Ellman *et al.* (1972) since such cells would be unable to induce a graft-versus-host reaction. The possibility that a graft-versus-host reaction may be responsible for the resistance induced by viable Hooded lymphoid cells was excluded by immunization of August rats with spleen cells from $F_1$ hybrids (August × Hooded). Such spleen cells cannot induce a graft-versus-host reaction in an August rat but carry Hooded antigens and will therefore induce immunity to such antigens. The finding (see Table I) that the protection provided by the $F_1$ (August × Hooded) spleen cells is as good as, if not better than, that induced by Hooded spleen cells is consistent with the view that the protection is due to the induction of specific immunity to an alloantigen of Hooded phenotype rather than stimulation of the host by a graft-versus-host reaction. Immunization with Wistar spleen cells and August × Marshall $F_1$ spleen cells was less effective in protecting against a challenge of SAL, suggesting that the Hooded "component" is more important than a host-versus-graft reaction. This is further confirmed by the reverse experiment in which $F_1$ August × Hooded rats immunized with either Hooded or August spleen showed no increased resistance to challenge with SAL. (SAL cells grow as well in August × Hooded $F_1$ hybrids as in August rats.) In this situation a graft-versus-host reaction should occur while there would be no immunological reaction to the cells used for immunization (either Hooded or August). The SAL rat leukaemia, therefore, differs from the guinea-pig leukaemia (Katz *et al.*, 1972; Ellman *et al.*, 1972) in which protection occurs only following inoculation with cells that can induce a graft-versus-host reaction.

*Serological evidence for cross reaction between the TSTA of SAL and a membrane antigen in Hooded cells*

The induction of resistance to SAL by a variety of normal and malignant Hooded cells is consistent with the hypothesis that the SAL cells express on their surface an antigenic determinant which is absent from the normal cells of adult August rats but which is present on many normal and malignant cells of Hooded rats. Since immunization of August rats with spleen cells from Wistar rats and August × Marshall $F_1$ hybrid rats does not evoke such strong immunity to SAL cells, we feel justified in referring to the antigen as Ho-SAL. While we were unable to demonstrate cytotoxicity to SAL cells using various sources of complement and the trypan blue exclusion method with antisera raised against SAL cells either in

* No protection against HRL cells injected into syngeneic Hooded rats could be induced with cells from Wistar or August rats.

syngeneic August rats or in Wistar rats following thorough absorption with normal August tissue, antibody was found in these sera as well as in the sera of Wistar rats immunized with Hooded cells (and then absorbed by August tissue) by indirect immunofluoresence and by migration inhibition (see Tables II and III).

In order to be sure that the binding of antibody to SAL cells is specific, it is necessary to remove from the test serum immune complexes or aggregated immunoglobulins since the SAL cells have receptors that bind complexes or aggregates. For this reason the alloantisera raised in Wistar rats which have been rendered specific for SAL by exhaustive absorption with normal August tissues had then to be freed of such complexes. This was done by absorbing the absorbed sera on Wistar myeloid cells, spleen cells and macrophages until the sera no longer bound to Wistar macrophages (Table IV).

Two types of absorbed allosera and a syngeneic hyperimmune serum were tested by immunfluorescence (see Table II):

(a) Sera from Wistar rats immunized with SAL cells after absorption against normal August tissues, *in vivo* and *in vitro*, would be expected to react only with

TABLE II.—*Detection of Ho-SAL by Indirect Immunofluorescence using Allo- and Syngeneic Sera*

| Target cells | Lowest concentration of absorbed antiserum staining more than 80% of target cells: Wistar anti-SAL | Wistar anti-Hooded spleen cell serum | August anti-SAL |
|---|---|---|---|
| August | | | |
| Leukaemia SAL | 1/160*† | 1/40*† | 1/40 |
| Thymocytes | <1/2 | 1/5 | <1/1 |
| Bone marrow cells | 1/5 | 1/5 | <1/1 |
| Macrophages | 1/5 | 1/5 | 1/2 |
| Hooded | | | |
| Leukaemia HRL | 1/160 | 1/80 | 1/20 |
| Thymocytes | 1/80 | 1/40‡ | 1/10† |
| Bone marrow cells | 1/80 | 1/40 | 1/20 |
| HSN (cultured) | 1/80† | 1/40‡ | 1/40† |
| Fibroblasts (cultured) | 1/80 | 1/40 | 1/40 |
| Foetal cells (cultured) | 1/5 | <1/5 | 1/20 |
| Macrophages | 1/80 | 1/40 | 1/20 |
| Wistar | | | |
| Thymocytes | <1/1 | <1/1 | |
| Macrophages | <1/5 | <1/5 | <1/5 |

* Following absorption with normal Hooded spleen cells, or thymocytes or bone marrow cells the titre fell to 1/5.
† Following absorption with SAL cells the titre fell to 1/5.
‡ Following absorption with SAL cells the titre remained unchanged.

TABLE III.—*Detection of Ho-Sal Antigen by Migration Inhibition using Allo-antisera**

| Migrating cell | Lowest concentration of absorbed allo-antisera which produced 30% inhibition of migration: Wistar anti-SAL serum | Wistar anti-Hooded spleen cell serum |
|---|---|---|
| August | | |
| Leukaemia SAL | 1/80 | 1/30 |
| Thymocytes | <1/5 | <1/5 |
| Hooded | | |
| Thymocytes | 1/100 | 1/80 |
| Wistar | | |
| Thymocytes | <1/5 | <1/5 |

* Normal August serum and normal Wistar serum tested at 1/5–1/80 dilutions did not produce inhibition of migration of greater than 5% compared with the foetal calf serum controls.

TABLE IV.—*Evidence for the Removal of Soluble Immune Complex from Test Antisera by Absorption against Wistar Rat Cells which Carry Fc Receptors*

| Sera tested | Lowest dilution giving membrane immunofluorescence on macrophage monolayers* | | |
|---|---|---|---|
| | Wistar | August | Hooded |
| Rabbit anti-rat IgG | 1/1 | 1/1 | 1/1 |
| Rat IgG | 1/40 | 1/40 | 1/40 |
| Ag/Ab complex‡ | 1/160 | 1/320 | 1/160 |
| After absorption† | 1/5 | 1/10 | 1/5 |
| Wistar anti-Hooded spleen cell serum | 1/80 | 1/160 | 1/160 |
| After absorption† | 1/5 | 1/5 | 1/40 |
| Wistar anti-SAL | 1/160 | 1/160 | 1/160 |
| After absorption† | 1/5 | 1/5 | 1/80 |
| August anti-SAL | 1/160 | 1/40 | 1/80 |
| After absorption† | 1/2 | 1/2 | 1/20 |

* Peritoneal exudate cells were cultured for 18 h and then treated with trypsin. Following addition of the test serum the presence of surface Ig was determined by indirect immunofluorescence with either a Goat anti-rat Ig FITC 1/30 or a goat anti-rabbit Ig FITC 1/60 conjugate (Nordic).

† Absorbed with bone marrow, spleen and peritoneal exudate cells and monolayers of Wistar macrophages.

‡ Produced by mixing at equivalence rat IgG with rabbit anti-rat IgG serum.

tumour specific antigens present in SAL cells. To obtain such a serum, the technique described by Weiner, Hubbard and Mardiney (1972) was used in which the SAL cells were coated before inoculation into Wistar rats with Wistar anti-August spleen cell alloantibody. This procedure, by reducing the immunogenicity of the normal alloantigens on the surface of SAL cells, facilitates the response to tumour antigens. This serum bound to (1) the membrane of viable SAL cells but not to any normal cells from August rats; (2) the membrane of all cells—normal and malignant—of Hooded phenotype. The fact that after absorption on normal Hooded cells this serum no longer combined with intact SAL cells indicates that the only tumour specific surface antigen of SAL which is detected by this sera cross reacts with a Hooded antigen. We have not been able to demonstrate that SAL cells have any additional tumour-specific membrane antigens which do not cross-react with Hooded alloantigens.

(b) Serum from Wistar rats immunized with normal Hooded rat tissue and then absorbed with normal August cells bound to the membrane of SAL cells but not to normal August cells. Absorption of this serum with SAL cells did not abolish its reactivity with normal Hooded cells. This is to be expected since it is probable that there should be several alloantigenic differences between Hooded and August cells.

(c) Serum from August rats immunized with mitomycin-C treated SAL cells was absorbed only with Wistar macrophages to remove putative immune complex and aggregated Ig. This serum bound to SAL cells and to Hooded cells but not to normal August tisses. Absorption on SAL cells abolished its activity against Hooded cells. This is again consistent with the concept that the tumour-specific membrane antigen of SAL cross-reacts with an antigen present on the membrane of normal Hooded cells.

Living SAL cells stained at 4°C with all these antisera show a very fine speckled appearance. The bound immunoglobulin caps when the temperature is raised to 37°C and capping is complete in 40 min when incubated with both the antibody and conjugate.

The measurement of migration inhibition (see Table III) by the 2 alloantisera is consistent with the hypothesis that the tumour-specific antigen on SAL is serologically identical to a membrane component present in normal thymocytes of

Hooded phenotype. Thymocytes were used as these behaved very reproducibly in the migration assay but this test cannot be used to establish the widespread representation of this antigen on different types of Hooded cells.

### DISCUSSION

Both the serological data and the induction of resistance by immunization with allogeneic Hooded cells suggest that the August rat leukaemia SAL carries on its membrane a determinant, the " Ho-SAL antigen ", which operationally behaves like a tumour-specific transplantation-type antigen (TSTA) to which the syngeneic host responds. Yet in the syngeneic situation immunization with SAL cells that have been rendered incapable of cell division by a variety of treatments induces a degree of host resistance which is very much less than that following immunization with cells of Hooded phenotype (*i.e.* compare Table I and data in Wrathmell and Alexander, 1976).

The fact that August rats but not August $\times$ Hooded $F_1$ hybrids can be immunized with Hooded cells against the syngeneic SAL leukaemia provides strong support for the view that the TSTA on SAL cross-reacts with an antigen of normal Hooded cells. Why then are Hooded cells much more immunogenic than SAL cells? We are clearly not dealing with a genetic defect (*e.g.* an Ir gene deficit) which prevents the August rat from responding to Ho-SAL, as is the case for some mouse strains which fail to react to the strong leukaemia-related antigen, X-1 (Sato *et al.*, 1973). Two mechanisms which are not mutually exclusive can be envisaged: (1) response to the Ho-SAL antigen requires the presence of another antigen, as was found to be the case for the TSTA of E.L.4. leukaemia cells (Gorer and Amos, 1946) and for certain non-H-2 transplantation antigens (Sanderson, 1964); (2) x-irradiation, and to a lesser extent treatment with mitomycin-C, may impair the immunogenicity of SAL with respect to the Ho-SAL antigen, as has been suggested for the TSTA of certain rat sarcomata (Proctor, Rudenstam and Alexander, 1974; Wrathmell and Alexander, 1976).

Ho-SAL has a formal similarity to TL, $G_{IX}$ and MM antigens in that they are tumour specific in some strains but are normal alloantigens in others. Ho-SAL, however, differs from these mouse antigens in, (1) being expressed not only on normal lymphoid cells but on other cells of Hooded phenotype; (2) not producing cytotoxic antibodies. $G_{IX}$ and MM are closely related to oncogenic viruses and are probably coded by viral genes present in the genome of most mice but only phenotypically expressed by some. There is no indication for a viral association of the Ho-SAL antigen at present and the biological basis for its selective expression based on phenotype remains to be determined. Experiments are in progress to determine whether the So-SAL antigen or other SAL-associated antigen is present on the membrane of other August rat tumours.

This investigation has been supported by Funds from the Leukaemia Research Fund and one of us (C. L. Gauci) is a Research Fellow of the Cancer Research Campaign.

### REFERENCES

BOYSE, E. A., OLD, L. J. & LUELL, S. (1963) Antigenic Properties of Experimental Leukaemias. II. Immunological Studies *in vitro* with C57 BL/6 Radiation Induced Leukaemias. *J. natn. Cancer Inst.*, **13**, 987.

BOYSE, E. A., OLD, L. J. & STOCKERT, E. (1965) The TL (Thymus Leukaemia) Antigen. A Review. In *Immunopathology*, IVth International Symposium. Ed. P. Grabar and P. A. Miescher. Basel: Schwabe and Co. p. 23.

CHANG, S., NOWINSKI, B. C., NISHIOKA, K. & IRIE, R. F. (1972) Immunological Studies on Mouse Mammary Tumours. VI. Further Characterisation of a Mammary Tumour Antigen and its Distribution in Lymphatic Cells of Allogeneic Mice. *Int. J. Cancer*, **9**, 409.

CURRIE, G. A. & SIME, G. C. (1973) Syngeneic Immune Serum Specifically Inhibits the Motility of Tumour Cells. *Nature, New Biol.*, **241**, 284.

ELLMAN, L., KATZ, D. H., GREEN, I., PAUL, W. E. & BENACERRAF, B. (1972) Mechanisms Involved in the Anti-leukaemic Effect of Immunocompetent Allogeneic Lymphoid Cell Transfer. *Cancer Res.*, **32**, 141.

Gorer, P. O. & Amos, D. B. (1956) Passive Immunity in Mice against C57 BL Leukosis EL4 by Means of Iso-Immune Serum. *Cancer Res.*, **16**, 338.

Invernizzi, G. & Parmiani, G. (1975) Tumour Associated Transplantation Antigens of Chemically Induced Sarcoma Cross-reacting with Allogeneic Histocompatibility Antigens. *Nature, Lond.*, **254**, 713.

Katz, D. H., Ellman, L., Paul, W. E., Green, I. & Benacerraf, B. (1972) Resistance of Guineapigs to Leukemia following Transfer of Immunocompetent Allogeneic Lymphoid cells. *Cancer Res.*, **32**, 133.

Kobayashi, H., Gotohda, E., Kuzumaki, N., Takeichi, N., Hosokawa, M. & Kodamat, A. (1974) Reduced Transplantability of Syngeneic Tumours in Rats Immunized with Allogeneic Tumours. *Int. J. Cancer*, **13**, 522.

Proctor, J. W., Rudenstam, C. M. & Alexander, P. (1974) A Preliminary Investigation into the Role of Immunity in Modifying the Blood Borne Spread of Chemically Induced Rat Sarcomas. *J. natn. Cancer, Inst.*, **53**, 1671.

Sanderson, A. R. (1964) Applications of Isoimmune Cytolysis using Radiolabelled Target Cells. *Nature, Lond.*, **204**, 250.

Sato, H., Boyse, E. A., Aoki, T., Iritani, C. & Old, L. J. (1973) Leukaemia Associated Transplantation Antigens Related to Murine Leukemia Virus. The X-1 System: Immune Response Controlled by a Locus Linked to H–2. *J. exp. Med.*, **138**, 593.

Stockert, E., Old, L. J. & Boyse, E. A. (1971) The GIX System: A Cell Surface Allo-antigen Associated with Murine Leukaemia Virus, Implications Regarding Chromosomal Integration of the Viral Genome. *J. exp. Med.*, **133**, 1334.

Weiner, R. S., Hubbard, J. D. & Mardiney, M. R. Jr (1972) Production of Tumor Specific Antibody in the Xenogeneic Host. Use of Blocking Antibody. *J. natn. Cancer Inst.*, **49**, 1063.

Wrathmell, A. B. (1976) The Growth Patterns of Two Transplantable Acute Leukaemias of Spontaneous Origin in Rats. *Br. J. Cancer*, **33**, 172.

Wrathmell, A. B. & Alexander, P. (1973) Growth Characteristics and Immunological Properties of a Myeloblastic and a Lymphoblastic Leukaemia in Pure Line Rats. In: *Unifying Concepts of Leukemia. Bibl. haemat.* No. 39. Ed. R. M. Dutcher and L. Chieco-Bianchi. Basel: Karger, p. 649.

Wrathmell, A. B. & Alexander, P. (1976) Immunogenicity of a Rat Leukaemia of Spontaneous Origin (SAL). *Br. J. Cancer*, **33**, 181.